# Motion of glossy objects does not promote separation of lighting and surface colour

**DOI:** 10.1098/rsos.171290

**Published:** 2017-11-15

**Authors:** Robert J. Lee, Hannah E. Smithson

**Affiliations:** 1 School of Psychology, University of Lincoln, Brayford Pool, Lincoln, LN6 7TS, UK; 2 Department of Experimental Psychology, University of Oxford, Oxford, UK

**Keywords:** vision, colour, material perception, motion, gloss

## Abstract

The surface properties of an object, such as texture, glossiness or colour, provide important cues to its identity. However, the actual visual stimulus received by the eye is determined by both the properties of the object and the illumination. We tested whether operational colour constancy for glossy objects (the ability to distinguish changes in spectral reflectance of the object, from changes in the spectrum of the illumination) was affected by rotational motion of either the object or the light source. The different chromatic and geometric properties of the specular and diffuse reflections provide the basis for this discrimination, and we systematically varied specularity to control the available information. Observers viewed animations of isolated objects undergoing either lighting or surface-based spectral transformations accompanied by motion. By varying the axis of rotation, and surface patterning or geometry, we manipulated: (i) motion-related information about the scene, (ii) relative motion between the surface patterning and the specular reflection of the lighting, and (iii) image disruption caused by this motion. Despite large individual differences in performance with static stimuli, motion manipulations neither improved nor degraded performance. As motion significantly disrupts frame-by-frame low-level image statistics, we infer that operational constancy depends on a high-level scene interpretation, which is maintained in all conditions.

## 1. Introduction

For our sense of vision to support our daily interaction with objects of the physical world, we must distinguish between different causes of variation in the retinal image—those that are due to the properties of objects and those that are due to the conditions of observing. As the spectral content of light reflected from an object depends not only on the spectral reflectance of the object but also on the spectral content of the illuminant [1], correctly identifying a particular object—the ripest fruit for example—depends on compensating for any differences in illumination. The image of a glossy surface can exhibit spatial variation in chromaticity owing either to surface patterning or to variation in the light field reflected from the surface. In principle, surfaces could be painted in such a way as to make them appear glossy, or highlights could be misidentified as surface patterning. How might observers disambiguate surface and lighting contributions to the image? One possible cue is that motion of the light sources or the surface will cause relative motion between patterning that belongs to the surface and spatial variation due to lighting. We know that observers’ judgements of glossiness are sensitive to such relative motion cues [2]. In this paper, we test whether operational colour constancy—the ability to distinguish colour changes caused by changes in the spectral reflectance of a surface from those caused by changes in the spectral content of the illuminant [3]—is influenced by relative motion between surfaces and specular reflections.

### 1.1. Background

Surface colour and surface patterning, dictated by pigmentation, can provide useful information about the identity or state of objects. This information is carried in the diffuse reflectance component of light reflected from the object. However, the vast majority of objects also offer a non-zero specular reflectance from their surface, reflecting features of the environment and light sources without spectral transformation, which can contribute to a glossy appearance [4]. As specular and diffuse reflection components are produced in different ways (see for example [5] and the Ward model [6]), they differ in their spectral and geometric properties, which might allow perceptual separation of these two components of an image. For non-white objects (those with a non-uniform spectral reflectance function), diffuse and specular components differ in their spectral composition. They also exhibit different imaging geometries. Variation owing to surface patterning is usually rigidly attached to the surface, whereas the positions of features in the specular component depend on the spatial arrangement of the surface, the light sources and the viewer. In natural viewing, these geometric relationships are rarely fixed. When judging real objects, the viewer may purposefully manipulate the object [7] and changes in the spectrum of the illumination, owing for example to the distribution of shadows, are typically accompanied by changes in illumination geometry [8]. In the following sections, we summarize the perceptual signals that are available from images of glossy objects, when illuminated by lights of different spectral composition and when stationary or moving, and consider how these signals might enable human observers to identify object properties from image properties.

### 1.2. Colour signals

**Figure 1.**
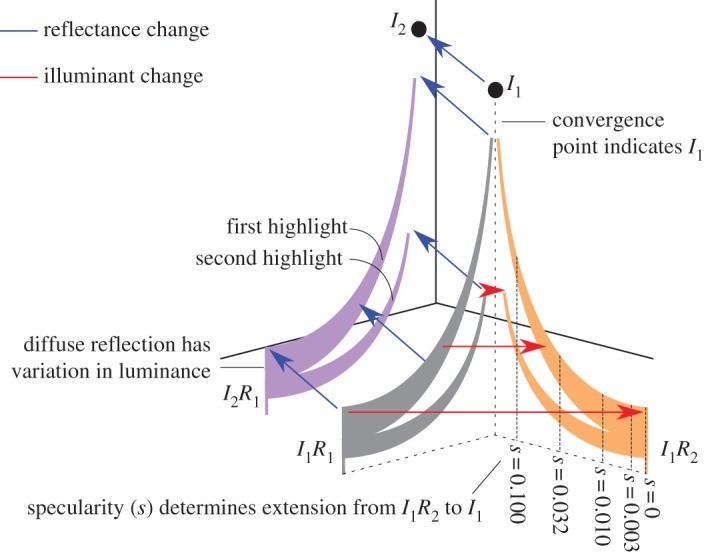
Cartoon showing the chromaticity changes that take place in an image containing a surface with a single reflectance lit by two point sources with the same spectra, when either the illuminant (*I*) or surface reflectance (*R*) changes. We use the MacLeod & Boynton [10] chromaticity diagram and a vertical axis to represent intensity. The chromaticities before either change are contained within the grey plane. Chromaticities in the diffuse reflectance are in the vertical distribution at *I*_1_*R*_1_. Chromaticities in the specular highlights are in the crescent shapes between *I*_1_*R*_1_ and *I*_1_, and each crescent contains the chromaticities from a separate highlight. A change in the spectral reflectance (*R*_1_ to *R*_2_) results in the transformation indicated by the red arrows, with the resulting chromaticities indicated by the orange plane. The diffuse reflectance chromaticities translate through colour space (*I*_1_*R*_1_ to *I*_1_*R*_2_), while the highlight chromaticities always extend towards the fixed point *I*_1_, and so chromaticities closer to that point translate less than those in the diffuse reflection. The surface specularity (*s*) determines how close to *I*_1_ the highlight chromaticities reach, and fine-dashed lines indicate these distances from *I*_1_*R*_2_. A change in the illuminant spectral power distribution (*I*_1_ to *I*_2_) results in the transformation indicated by the blue arrows, with the resulting chromaticities indicated by the purple plane. All chromaticities translate through colour space by a similar amount, maintaining the relative positions of the diffuse reflection chromaticities (*I*_1_*R*_1_ to *I*_2_*R*_1_) and illuminant chromaticities (*I*_1_ to *I*_2_).

Previously we showed that non-zero specularity can support operational colour constancy with single surfaces, and that observers perform better as specularity increases [9]. The signals underpinning this performance derive from the chromatic variations across the image of a surface that are introduced by specular reflections (figure 1). Matte surfaces, with zero speculatrity, reflect only the diffuse component, whose spectral content is a wavelength-by-wavelength multiplication of the surface reflectance function and the illuminant spectrum (*I*(λ)×*R*(λ)). Conversely, with high specularity, some regions are almost completely dominated by the specular reflection, which carries the spectral content of the illuminant (*I*(λ)), and with low specularity, the reflected light, at least for most non-metallic surfaces, is constrained to be a linear mixture of the specular and diffuse components (i.e. *aI*(λ)+*bI*(λ)×*R*(λ), where *a* and *b* are scale factors that depend on the imaging geometry). A change in the illuminant will cause the colour of both diffuse and specular components to change similarly, while a change in the surface spectral reflectance will cause the specular highlights to change colour less than the parts of the image that are dominated by diffuse reflection (figure 1). The chromatic signature from a reflectance change is the temporal analogue of chromaticity convergence [11], which has long been identified [12,13] as a cue to the illuminant, because chromaticities in the image of a glossy surface with two or more spectral reflectances lie on lines in colour space that intersect at the illuminant chromaticity. Importantly, chromaticity convergence provides information about the illuminant even at low specularities when the illuminant chromaticity is not directly available in the image. However, chromatic statistics alone are not sufficient to explain operational colour constancy. Lee & Smithson [9] showed that phase-scrambled stimulus images—in which the chromatic statistics were preserved, but the spatial structure of the scene could not be inferred—led to a marked deterioration in performance.

### 1.3. Interactions between motion, texture, shape and glossiness

Whereas, for diffuse reflections, light is scattered in every direction from the surface, for specular reflections, light is reflected at the same angle from the normal at which it is incident, with usually a smaller amount of scattering that depends on the roughness of the surface. These differences in imaging geometry have important consequences for the relationship between material properties, such as texture (variation or patterning in surface reflectance), colour and glossiness, and the proximal image, and for the transformations imposed by shape and motion. For example, with static stimuli, texture information (conveyed in the diffuse component) gives rise to the compressions in the proximal image that depend on surface orientation (the first derivative of surface depth), whereas specularities generate compressions that depend on surface curvature (the second derivative of surface depth), leading to distinctive orientation fields in the image that can be used to derive shape information [14]. The perception of texture can be reduced if the orientation of lightness changes created by the texture are consistently aligned with shading created by surface relief [15], and the perception of gloss can be reduced if highlights are rotated or translated so that they are inconsistent with the lightness variation in the diffuse reflectance [16].

In general, the virtual image of a light source, a specular highlight, is located in depth behind a glossy convex surface and in front of a concave surface. Human observers are sensitive to the stereoscopic appearance of highlights, which affects both the perception of surface curvature and the appearance of glossiness [17,18,19]. Similarly, relative motion of the surface, lights or viewer, is likely to cause highlights to move relative to the surface, and such motion cues influence judgements of shape [20,21,22,23] and glossiness [24,25,26]. Proximal image features—such as size, contrast, sharpness of highlight regions—can enhance glossiness, regardless of whether it is the surface geometry, light field, or specularity that actually generates the structure [27]. Importantly, three-dimensional shape constraints (conveyed by stereospsis or by shape-from-motion or shape-from-texture) do influence perception of glossiness in stimuli that contain identical luminance gradients [28,29,30]. In a particularly compelling demonstration, Doerschner *et al.* [2] show that movies displaying standard specular motion of a specular object rotating back and forth promote a glossy appearance, whereas movies in which the reflections were rigidly attached to the surface promote a matte appearance. These studies confirm that information which is not available in a monocular static image can have a strong effect on separating surface and lighting contributions to the proximal image.

Multiple views of light reflected from a surface can provide enhanced information about the geometry of the light sources [31,32]. We might then expect these factors to affect colour constancy, because there is evidence that lightness and colour perception are affected by the observer’s interpretation of the three-dimensional structure of the objects and lighting in a scene [33,32,34,35,36,37,38,9]. Although we are primarily interested in operational colour constancy, the effects of motion on shape estimation are particularly strong, and cues to surface shape may have indirect effects on surface colour perception. Motion of a patterned, matte object can provide rich information about surface shape. In shape-from-motion experiments [39,40,41,42], dots whose motion is consistent with them being attached to a rigid, moving surface, are projected onto a two-dimensional display. Observers integrate the consistent motion of the dots and perceive the three-dimensional surface to which the dots are attached, whereas without the motion they see only randomly positioned dots. When a patterned, glossy surface moves, analogous shape-from-motion signals are carried in the optic flow of the diffuse component of the image. However, specular flow carries information about second-order shape properties [43], and its exact contribution to shape estimation has been debated. While it may disrupt shape estimation in some cases [22], it has also been shown to enhance estimates of three-dimensional curvature, dominating cues from optic flow of surface pattern [44]. Information carried in optic flow of the diffuse component can be dissociated from specular flow by comparing rotations of an object around a vertical axis with rotations around the viewing axis, a manipulation we use in the present experiments. While both rotations preserve the curvature information, only rotation around a vertical axis preserves information conveyed by optic flow about surface slant [45].

There is now strong evidence to suggest that motion influences perceived glossiness. There have, however, been fewer studies testing the effects of motion on perception of other material properties, such as lightness and colour, with glossy objects. Wendt *et al*. [26] asked participants to match the perceived lightness and glossiness of two surfaces with different shapes, and manipulated cues that may help separate reflectance and shape properties. Information from motion, binocular disparity and colour all improved the constancy of glossiness matches, both in isolation and in combination. Lightness constancy, however, was affected only by motion and colour information, and for motion the effects were counterintuitive, with motion impairing performance. When a surface has a complex shape, highlights appear and disappear or change size and shape as the local orientation of the surface to the observer and light source changes. Such disruption of the image might impair access to surface features signalling lightness, and we might expect it to also impair colour constancy tasks.

### 1.4. Rationale

Correctly parsing an image to identify the objects and illumination that produced it is a mathematically under-constrained problem. As summarized above, motion of glossy objects has been shown to have a strong impact on their perceived glossiness and their shape, suggesting that observers are sensitive to the geometric differences between diffuse and specular components of an image. For operational colour constancy, relative motion between patterning that belongs to the surface and spatial variation owing to specular reflections might provide a cue to separate the diffuse and specular reflectance components of an image. However, the fine chromatic discriminations that are required in operational colour constancy may be more difficult in the presence of motion that causes the most informative regions of the image (those associated with highlights) to move, at best, or to be scrambled, at worst, making them harder to track.

To assess human observers’ sensitivities to these competing factors, we presented them with animations of isolated glossy objects lit by discrete sources and measured their operational colour constancy, as a function of surface specularity, under different conditions of motion. Stimuli were designed to isolate the specularity cue to operational colour constancy. In a scene comprising many surfaces under one illuminant, there are several image properties—such as the mean chromaticity—that correlate with the illuminant chromaticity. But, for the stimuli used here, illumination and reflectance could be separated only by accessing information conveyed in the specular component of the image. In a factorial design, we compared performance for two types of object, two sources of motion and two axes of motion.

The two types of object were chosen to manipulate the relative patterns of motion of the diffuse and specular components in the image. One object was a smooth sphere, with a surface pattern (marbled); the other object was a bumpy sphere, with no surface pattern (bumpy). As surface patterning and bumpiness were never present on the same object, we do not test interactions between pattern and shading [15]. For the smooth marbled sphere, some motion conditions caused the highlight regions to move, but their spatial layout was never scrambled. Conversely, for the bumpy sphere, all motion conditions caused highlight regions to be scrambled.

We compared constancy performance when stimuli included motion of the object or of the lights. These sources of motion allowed us again to compare different patterns of highlight motion. For the marbled sphere, the two motion conditions had different effects on the highlights: motion of the (radially symmetric) object had no effect on highlight locations, though the surface pattern was displaced; motion of the lights caused the highlight locations to move, though the surface pattern remained stationary. For the bumpy sphere, both motion conditions disrupted the highlight locations and introduced changes in intensity (shading) of the diffuse component.

Finally, we chose to use two different axes of rotation that allowed us to manipulate the shape-from-motion information that was available from the diffuse component of the animation. A vertical axis, perpendicular to the viewing axis, provided typical shape-from-motion information. An axis aligned with the viewing axis was chosen to provide significantly less shape-from-motion information than given by rotation about the vertical axis, because points on the surface of the object move laterally across the image, rather than in depth (e.g. [44]).

We compared performance in these motion conditions with performance in two control conditions. In one control condition, there was no motion (equivalent to experiment 2 in [9]). In the other control condition, both the object and the light sources rotated about the visual axis (VA), equivalent to rotating the image on the screen. Neither control condition included relative motion of the diffuse and specular components of the image, but the second did include motion, and therefore provided a comparison for performance in conditions in which the locations of the most informative regions of the image moved.

## 2. Material and methods

### 2.1. Overview

Stimuli were computer-generated animations of a single, spherical object in a void, lit by three small light sources. At any instant, the surface had a single spectral reflectance (patterning for marbled stimuli changed reflectance by a scale factor only) and all three light sources shared the same spectral composition. Over the course of the animation, either the surface spectral reflectance (*R*(λ)) changed, or the illumination spectral power distribution (*I*(λ)) changed, but not both. It was the observer’s task to decide which had changed. Objects were rendered with one of five levels of specularity (specifying the proportion of light reflected in the specular component), from perfectly matte (*specularity*=0) to glossy (*specularity*=0.1). For matte stimuli, the operational colour constancy task is impossible, but with increasing specularity the signals available to solve the task increase. In different conditions of the experiment, we compare the rates of performance increase with increasing specularity.

### 2.2. Stimuli

**Figure 2.**
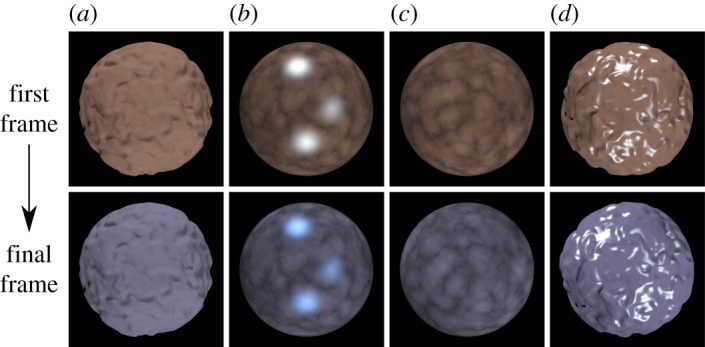
Examples of a subset of the stimuli used in our experiments. The top row shows the first frames and the bottom row shows the final frames from four animations. (*a,d*) Bumpy stimuli; (*b,c*) marbled stimuli. (*a,b*) No-Motion condition; (*c,d*) Image_VA_ condition. Combinations of illuminants and reflectances in this figure are chosen so that the diffuse reflection colour changes (visible in the zero specularity stimuli, (*a,c*)) are identical for illuminant and reflectance changes ((*a,c*), respectively). With non-zero specularity (*b,d*) the specular component supports operational colour constancy, identifying (*b*) as an illuminant change, and (*d*) as a reflectance change. Colour reproduction in this figure will not be accurate.

Images were rendered using hyperspectral raytracing with RADIANCE [46] and custom routines. The object was either a sphere whose surface had been modified by displacing the surface depth according to procedural noise (Blender’s marble texture, Blender Foundation, Amsterdam, The Netherlands), or a sphere with reflectance intensity modified by a volumetric turbulence function (RADIANCE’s marble function). We refer to these as ‘bumpy’ and ‘marbled’ spheres, respectively, and they are the same as the objects used in experiment 2 of our previous study [9]. The rendered stimuli were converted to a 14-bit per pixel per channel RGB image for display on a CRT monitor driven by a Cambridge Research Systems (Rochester, UK) ViSaGe. Examples are shown in figure 2.

The light sources were assigned spectral power distributions (*I*(λ)) of either sunlight or skylight [47] and, in any one frame, all three sources had the same spectral distribution. The reflectance of the surface was specified with a spectral reflectance function (*R*(λ)) and a specularity value. Spectral reflectances were selected from a database of measurements of natural and man-made surfaces [48,49,50,51] (also see spectra.csv in the electronic supplementary material). As we used only two illuminants, an arbitrary selection of spectral reflectances would permit above chance performance in operational colour constancy based solely on the direction and magnitude of chromatic change of the diffuse component of the image: changes that were not aligned to the blue-yellow direction would be more likely to be surface changes. Therefore, to silence this cue, pairs of reflectances were selected such that the distributions of the direction and magnitude of chromatic change of the diffuse component were matched for the surface-change and illuminant-change trials.

In any given trial, specularity (as defined by RADIANCE’s plastic definition) was fixed at one of five values: zero and four logarithmically spaced values from 10^−2.5^ to 10^−1^. The maximum value of specularity we used (10^−1^) is a realistic value for a material of this kind [6] and appears glossy. Lower values produced materials with a slight sheen, and materials with very low specularities appeared matte. The maximum specularity is high enough that the brightest pixels were close to the illuminant chromaticity, while, for lower specularities, the light from the brightest points in the image contained a mixture of specular and diffuse components. The roughness parameter was fixed at 0.15 for all our stimuli.

The stimulus for each trial comprised a short animation, showing either a surface reflectance change, or an illuminant spectral change. The animation was either accompanied by motion or not, as specified in the following section. The dynamic part of each animation lasted 0.33 s, with additional static periods at the beginning and end, each lasting 0.50 s.

### 2.3. Motion conditions

**Figure 3.**
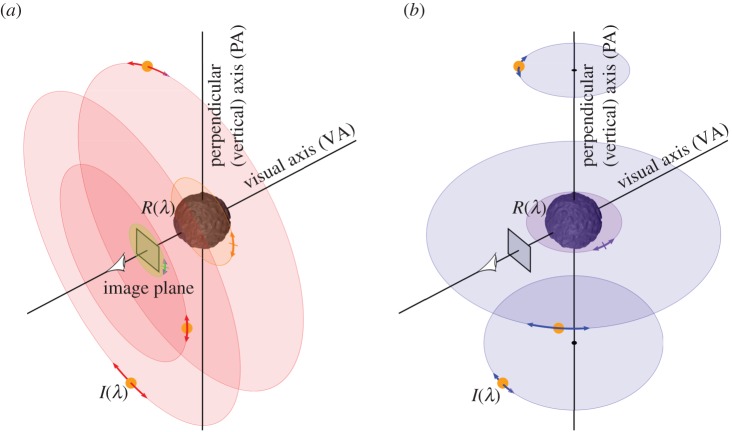
The virtual scene used to generate our stimuli, not shown to scale. During the animation either the spectral reflectance of the surface (*R*(λ)) or the spectral power distribution of the illuminant (*I*(λ)) changed over 0.33 s. In separate experimental conditions, either the rendered image (indicated by a rectangular plane), the object (shown at the intersection of two axes), or the three light sources (indicated by orange points) rotated by ±30° around one of two axes, at the same time as the spectral change. (*a*) Rotations of the image (green arrows), light sources (red arrows) and object (orange arrows) around the visual axis (VA). (*b*) Rotations of the light sources (blue arrows), and object (purple arrows) around the perpendicular axis (PA). Lightly coloured discs identify the planes in which rotations occurred.

**Figure 4.**
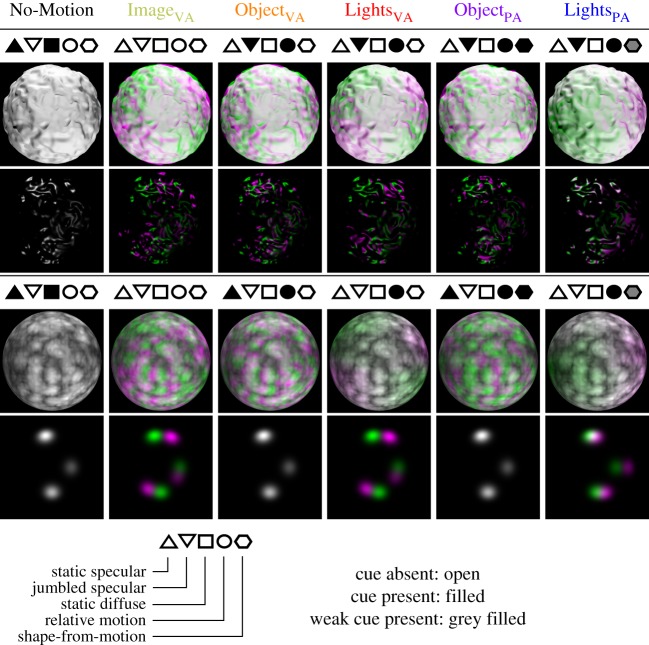
The motion-related signals present in each condition. Motion conditions No-Motion, Image_VA_, Object_VA_, Lights_VA_, Object_PA_ and Lights_PA_ are presented in different columns, and bumpy and marbled stimuli are represented in the upper and lower panels. For each condition, we present separately the diffuse and specular components of the stimuli, in adjacent rows. Within each image, the intensity of light in each component is coded in the green (G) channel for the first frame and in the magenta (*R*+*B*) channel for the last frame. Where the first and last frames match, a greyscale (*R*+*G*+*B*) image is produced. In the No-Motion condition, the first and last frames match perfectly. For marbled stimuli, object rotation (Object_VA_ and Object_PA_) has no effect on the specular component which remains stationary. For all other conditions, the green and magenta regions of the images show changes in the diffuse and specular components between the first and last frames. For Image_VA_, both the diffuse and specular components undergo the same rigid rotational transformation. Rotation of the lights (Lights_VA_ and Lights_PA_) produces broadly the same change in the diffuse components of bumpy and marbled stimuli, determined by the spherical shape of the stimulus. Rotation of the objects (Object_VA_ and Object_PA_) produces changes in the diffuse components that convey shape-from-shading information and patterning, respectively. The specular component of bumpy stimuli is more complex than the specular component of marbled stimuli, and the highlight pattern is disrupted by motion of either the object or of the lights.

For bumpy and for marbled stimuli, motion of the lights or of the object could be around the VA or a vertical axis, perpendicular to the VA (PA). We refer to these conditions as Lights_VA_, Object_VA_, Lights_PA_ and Object_PA_. In control conditions, there was either no motion, or motion of the image about the VA. We refer to these conditions as No-Motion and Image_VA_. We note that the ‘visual axis’ was that of the viewpoint from which the scene was rendered, not strictly that of the observer, who was free to move his/her gaze. We illustrate the scene geometry and rotation axes in figure 3. With six motion conditions (four, plus two control conditions), and two types of object (bumpy and marbled) we have 12 experimental conditions in total (figure 4).

In all cases, the direction of rotation about the axis was selected randomly to be clockwise or anticlockwise by 30°. The rotational motion occurred only during the 0.33 s period of animation that included the spectral change, had constant rotational speed (90° *s*^−1^) and abrupt onset and offset. Linear motion of points in the image corresponded to a maximum speed of 5.6° *s*^−1^ (degrees of visual angle per second) at the outer edge of the sphere for the VA conditions and 21.3° *s*^−1^ (degrees of visual angle per second) across the centre of the sphere for the vertical-axis conditions. Examples of initial and final frames from our stimulus animations are shown in figure 2.

### 2.4. Procedure

The observer’s task was to classify each animation as a surface change or an illuminant change. Responses were collected via a button box. Observers viewed the computer-generated stimuli monocularly (to remove conflicting binocular disparity cues, which show the image to be in a single plane), in a dark room, with a black cardboard viewing tunnel to restrict stray light. At the viewing distance of 1.0 m, the stimuli subtended a visual angle of 7.1°×7.1°.

We ran the 12 experimental conditions in separate experimental sessions, each comprising 700 unique trials (140 at each of five levels of specularity), which were divided into four blocks of 175 trials, each block lasting approximately 15 min. The order in which the conditions were run was counterbalanced.

### 2.5. Observers

Six observers (1–6) participated in this experiment after giving their informed consent in accordance with the University of Oxford ethical procedures. All observers completed all conditions (except observer 3 who was not available to complete the conditions with rotation of the object or light sources about a vertical axis, Object_VA_ and Lights_VA_). All observers had normal colour vision (no errors on the HRR plates and a Rayleigh match in the normal range measured on an Oculus HMC-Anomaloskop), and normal or corrected-to-normal visual acuity. Observers 4 and 6 are male; all others are female. Observer 4 is one of the authors; observer 6 was aware of the purpose of the experiment; the other observers had received an undergraduate psychology course on perception, but were naive to the purposes of the experiment. Observers 1, 2 and 4 participated in a previous study with similar stimuli [9] (as observers 3, 4 and 1, respectively). All participants had an opportunity to practise the task, except observer 6, whose early sessions were discarded.

## 3. Results

### 3.1. Performance per observer

The raw responses from observers are provided in the attached spreadsheet (see responses.csv in the electronic supplementary material). From these data, we calculate performance in discriminating between reflectance and illumination changes. We use *d*^′^ and ln(*β*) to independently indicate sensitivity and response-bias, respectively. A *d*^′^ value of 0 indicates that performance was at chance level, while the maximum measurable *d*^′^ from 140 trials is approximately 4.9 (because 140×*p*(*z*<(4.9/2))=140×0.9928=139). A ln(*β*) of 0 indicates no response bias, while a positive value indicates a tendency to give a ‘reflectance change’ response and a negative value indicates a tendency to give an ‘illuminant change’ response.

**Figure 5.**
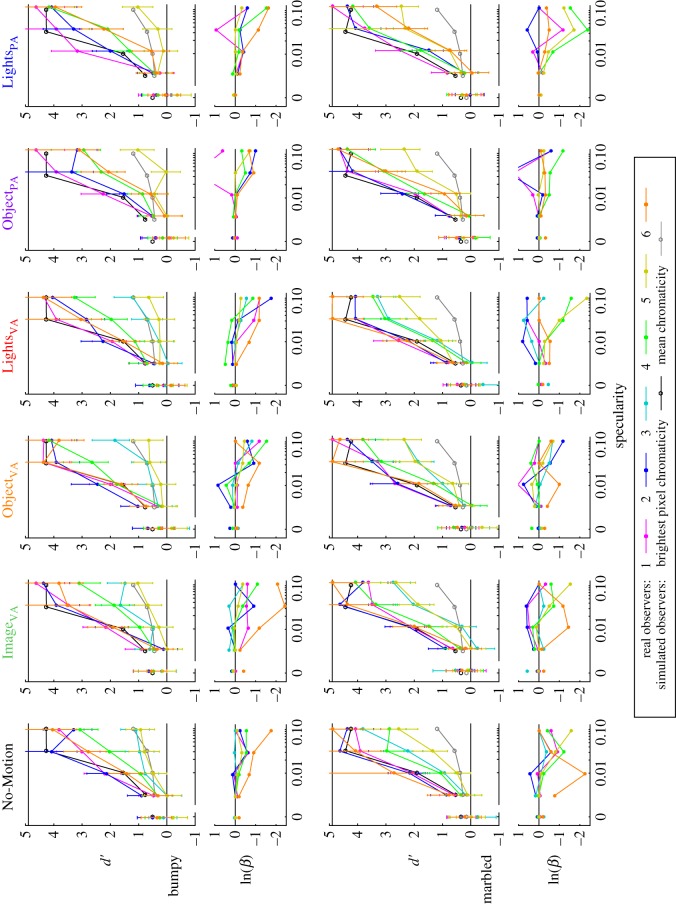
Results from our experiment, shown separately for each observer in each condition. Results from the bumpy surfaces are shown in the top row, and results from the marbled surfaces are shown in the bottom row. Results from the different motion conditions are shown in different columns. In each pair of plots, the upper plot shows *d*^′^ as a function of specularity, with results from each observer represented by a different coloured line. Error bars indicate 95% CI. The lower plot shows corresponding ln(*β*) values. The black curve shows mean results from a simulated observer who made judgements based on the brightest pixel in the image, and the grey line shows results from a simulated observer who made judgements based on the mean chromaticity of the image. These simulations are described in the text (§4.4). The black horizontal line at the top of each *d*^′^ plot indicates the maximum measurable value.

Performance for each observer in each condition—bumpy and marbled for each of six motion conditions—is shown in figure 5 as a function of specularity. As expected, performance (*d*^′^) was close to chance level with zero specularity and increased with specularity for all conditions and for all observers.

Performance with marbled stimuli was consistently higher than for bumpy stimuli, both in the no-motion conditions and in all moving conditions. These differences were particularly apparent at the highest specularities, but there is a performance advantage of between 30% and 60% for marbled stimuli throughout the range.

Observers 3–6 show less improvement with specularity for the bumpy surfaces than the marbled surfaces, and this difference is particularly pronounced in observers 3 and 5 who show less improvement than the other observers in general. The levels of performance attained are similar across conditions for a given observer, and ranking of observers’ performances is largely conserved.

There are no strong indications that the overall level of performance or the patterns of improvement with specularity are influenced by motion condition. Differences between observers also indicate that not all observers have reached a hard-limit of performance with these stimuli: even observers who perform poorly with stationary stimuli show no improvement in motion conditions.

An ANOVA on the *d*^′^ values, with surface type, motion condition and specularity as repeated-measures factors, reinforces these observations. None of the effects involving rotation type are statistically significant, and the only significant effects are those of surface type (*F*_1,4_=7.94,*p*=0.048), specularity (*F*_4,16_=45.04,*p*<0.01), and their interaction (*F*_4,16_=6.70,*p*<0.01).

Some observers in some conditions show ln(*β*) values that become increasingly negative at higher specularities. This is particularly noticeable for observers 4 and 5, but is not consistent across all observers or experimental conditions and estimates of ln(*β*) have larger error at high values of *d*^′^ because they are estimated from low numbers of false alarms.

### 3.2. Relative performance between conditions

**Figure 6.**
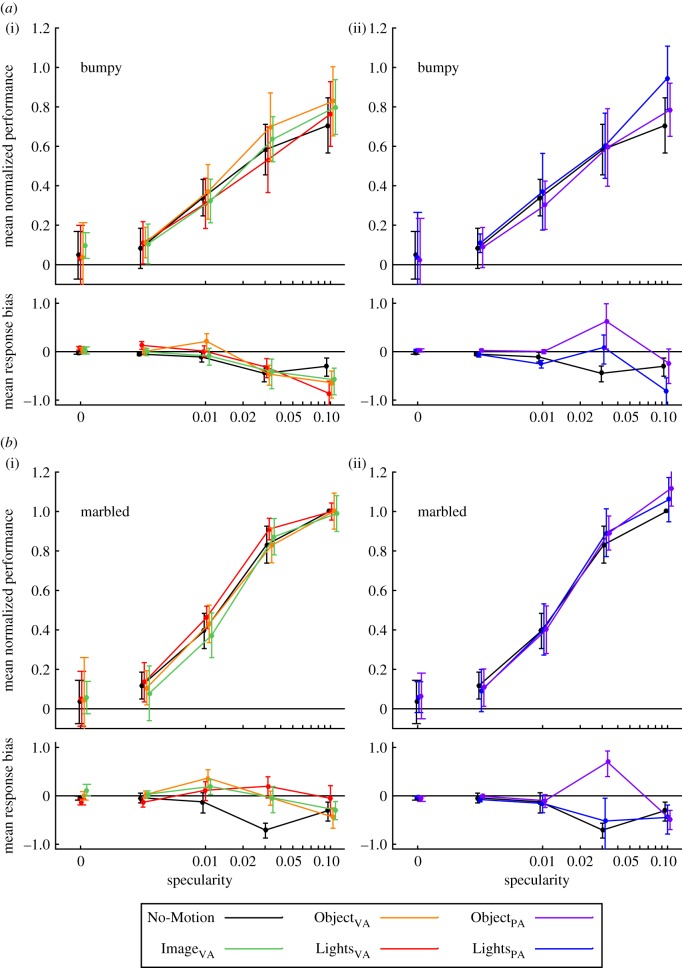
Summarized results from our experiment. The upper plot in each pair shows mean relative *d*^′^ values over all observers at each measured value of specularity. Before averaging, data from each observer were normalized to their performance with static marbled stimuli. The lower plot in each pair shows the corresponding mean ln(*β*) values. (*a*) Results from conditions in which observers viewed bumpy stimuli; and (*b*) results from conditions in which observers viewed marbled stimuli. (*a*)(i) and (*b*)(ii) Results from conditions in which there was no rotation (black), or rotation around the visual axis (VA) of the image (green), light sources (red) or the object (orange). (*a*)(ii) and (*b*)(ii) Results from conditions in which there was rotation around the vertical axis (perpendicular to the VA (PA)) of the light sources (blue) or the object (purple). Each curve is slightly displaced horizontally to allow comparison of error bars.

To facilitate comparison between conditions, figure 6 shows average relative performance for the 12 conditions of the experiment. Figure 6*a* shows performance with bumpy stimuli and figure 6*b* shows performance with marbled stimuli. Figure 6*a*(i),*b*(i) shows data from conditions in which motion was about the VA: Image_VA_ (green), Lights_VA_ (red) and Objects_VA_ (orange), with No-Motion (black) for comparison. Figure 6*a*(ii),*b*(ii) shows data from conditions in which motion was about the PA: Lights_PA_ (blue) and Objects_PA_ (purple), with the data from condition No-Motion re-plotted to provide a reference. Before averaging, *d*^′^ values for each observer were scaled relative to that observer’s performance with static marbled stimuli (which therefore plots at a normalized value of one in the graph, with no error).

Average performance is highly similar for all motion conditions. There are no cases in which data points at a given level of specularity separate beyond the measurement error for different motion conditions. Considering the curves as a whole, there is some suggestion that observers under-perform in the image-rotation condition (Image_VA_) at low specularities for marbled stimuli, but any advantage provided by relative motion (Lights_VA_ and Objects_VA_) is no greater than the advantage for stationary stimuli (No-Motion). For rotations around the PA (Lights_PA_ and Objects_PA_), performance is indistinguishable from the No-Motion condition, except for a small advantage at the highest specularities for both bumpy and marbled stimuli. Estimates of ln(*β*) are close to zero for most conditions, except for a reliable trend towards negative values (indicating more ‘illuminant change’ responses) for bumpy stimuli when rotation was around the VA.

Performance with marbled stimuli is consistently higher than for bumpy stimuli, both in the No-motion conditions and in all moving conditions. These differences are particularly marked at the highest specularities, with a performance improvement of between 25% and 50% for marbled stimuli.

## 4. Discussion

### 4.1. Operational colour constancy improves with increasing specularity

Observers perform better at distinguishing between illuminant and reflectance changes with increasing surface specularity, as they did with similar stimuli in a previous experiment [9]. The information available to support these judgements is contained in the spatio-chromatic properties of the stimuli. As discussed in §2.1, the structured chromatic changes produced by illuminant changes or by reflectance changes are characteristically different. For an illuminant change, both diffuse (*I*(λ) ×*R*(λ)) and specular (*I*(λ)) components change in chromaticity, while for a reflectance change, only the diffuse component is affected. At low specularities, chromaticities in the image are weighted mixtures of the diffuse and specular components and there may be no region of the image that is dominated by the specular component, so the illuminant chromaticity may not be directly available in the image. Nevertheless, observers perform above chance, even for low levels of specularity, suggesting that they are able to perceptually separate changes in the specular and diffuse components of the image.

We designed the stimuli so that the cues that are available in operational colour constancy tasks with multiple surfaces, such as ‘pop-out’ of inconsistently changing surfaces [52], are not available. However, for our stimuli, illuminant changes impose a single multiplicative transformation on cone excitations from pixels within the object, whereas reflectance changes introduce different transformations of diffuse regions and those regions that additionally include a non-zero specular component [9], fig. 3.

### 4.2. Inter-observer differences

We see considerable differences in operational constancy shown by the six observers. This replicates the inter-observer differences we measured previously [9]. The results of the two studies show a large variation in performance on this task between individuals. Other researchers have also found individual differences in performance in lightness constancy experiments [37], colour constancy [53,54,55] and gloss perception [26].

Importantly, although absolute performance differs between observers, we do not see consistent differences in *relative* performance between the experimental conditions (figure 6): Whatever an observer’s level of performance in the No-Motion condition, performance is neither enhanced nor impaired in the different motion conditions. The inter-observer differences in absolute levels of performance rule out floor or ceiling effects in the range of performance that can be attained with our stimuli and task, which might have masked the effect of motion.

### 4.3. Motion and perceptual grouping

Motion can certainly influence colour perception of illuminated surfaces. For example, colour constancy has been shown to be better with simple patch-like stimuli when they move against a background [56], perhaps because motion increases the saliency of the target patches. A perceptual-grouping task shows that colour-based segmentation is sensitive to facilitation or inhibition by motion-based segmentation signals, implying that the establishment of motion-based surfaces can form important primitives for further colour processing [57]. Movement of a transparent filter in front of a variegated surface allows perceptual separation of the chromatic properties of the surface and of the transparent filter itself [58]. In general terms, the motion-dependent enhancement in these cases can be attributed to the perceptual organization that motion imposes on the elements of the scene [59]. Perhaps, the most striking example of perceptual organization imposed by motion of shiny surfaces is the transition from a matte to glossy appearance that is triggered when relative motion is introduced between patterning that belongs to the surface and spatial variation due to lighting [2]. All experimental conditions in the present experiment (motion of lights or objects, for marbled or bumpy stimuli, about either axis of rotation) introduced relative motion between the diffuse and specular components of the image. Yet, none of these manipulations improved operational constancy relative to the No-Motion or Image_VA_ control conditions. This suggests that even the static images contain sufficient structure to support appropriate perception of the scene, and that observers who performed poorly were not limited by an inappropriate perceptual organization of the scene.

### 4.4. Chromatic discriminations

With low specularity stimuli, distinguishing reflectance changes from illuminant changes requires very fine chromatic discriminations, and would be limited by the overlapping distributions of chromatic change that are produced when environmental spectral reflectances and illuminants are used. In the matte (zero specularity) case, we have designed our stimuli to contain no reliable signal to support operational constancy, and indeed all observers perform at chance in these conditions. One way to quantify the increase in performance that might be expected as specularity is increased is to construct a model observer who has perfect colour discrimination and bases their decisions on a candidate image statistic, such as the chromaticities of the brightest pixels or the mean chromaticity of the image [9]. Such models predict increases in performance with increasing specularity, but the improvement is much greater for the brightest-pixel model than for the mean-chromaticity model, as shown with black and grey lines, respectively, in figure 5. For the No-Motion condition, the levels attained by an observer using the brightest pixels are comparable with the levels attained by the best observers.

The range of performance levels attained by different observers in this task shows that many of our observers (1, 2, 4 and 6) are not limited by the chromatic statistics that are, in principle, available, and yet they still show no performance enhancement with relative motion between specular and diffuse components. Additional cues to allow these components of the image to be disentangled, and cues that enhance the ‘realism’ of the rendered scenes, do not help. This suggests that the separation of the surface and lighting contributions to the image is not what is limiting their performance. The observers who perform relatively poorly in all conditions may simply have poorer chromatic discrimination ability and so be less able to access the chromatic signals required to discriminate specular and diffuse regions of the stimuli, regardless of any motion, but we do not directly test this.

For the remaining observers, performance is not impaired by disruption of image locations that carry most information in terms of chromatic change, nor is there any evidence that the motion itself impairs the detection of chromatic changes [60] that are critical to the task.

### 4.5. Motion and unpredictability

Not all locations in the image are equally informative for the operational constancy task. Pixels that are dominated by the specular component provide useful information, because they change most under an illuminant change, and image regions where specular and diffuse components are mixed to varying extents produce the chromatic gradients that form the basis of the chromaticity convergence cue [11]. However, as the observer is not presented with the diffuse and specular components separately, and instead sees only the composite image, the informative image regions must be identified from the image properties.

A consequence of the motion in our stimuli is reduced predictability in the locations of the useful chromatic information. In the No-Motion condition, the useful chromatic gradients remain in the same regions of the image for the duration of the animation. The Image_VA_ condition served as a control that contained motion, but no relative motion between the highlights and the surface. We saw no differences in performance between these conditions, suggesting that the smooth displacements of the relevant image-regions did not impair discrimination performance relative to the static case. This was true both for bumpy stimuli, where variation in the specular component is carried in relatively high spatial frequencies, and for marbled stimuli, where the specular variation is at lower spatial frequencies.

The effects of motion on the locations of useful chromatic information are different for bumpy and marbled stimuli. With bumpy stimuli, small regions of highlight appear and disappear as local regions of curvature change orientation with respect to the light sources [2]. If this disruption were catastrophic—perhaps by preventing observers from predicting and tracking relevant image-regions—we should see poorer performance in conditions in which the lights or bumpy object moved (Lights_VA_ and Object_VA_, and Lights_PA_ and Object_PA_), relative to performance in the image-rotation condition (Image_VA_ or No-Motion), but we did not.

With marbled stimuli, rotation of the object (Object_VA_ and Object_PA_) holds stationary the high intensity regions of the specular image component (see 3rd and 5th columns, bottom row of figure 4), whereas rotation of the lights (Lights_VA_ and Lights_PA_) causes these high-intensity regions of the specular component to move (see 4th and 6th columns, bottom row of figure 4). In both cases, there is relative motion between the texture pattern (carried in the diffuse component) and the specular component. For marbled stimuli, neither the relative predictability of the locations of highlights (Object_VA_ and Object_PA_, relative to Lights_VA_ and Lights_PA_), nor the relative motion of the two components (Lights_VA_, Object_VA_, Lights_PA_ and Object_PA_, relative to Image_VA_ or No-Motion), affects performance.

### 4.6. Texture introduces brightness variability

As noted above, one cue to identifying informative regions of the image is to use the brightest regions. The specular component alone contains large variations in intensity, which means that for high specularities bright regions in the composite stimulus image are likely to be dominated by the illuminant chromaticity. However, the marbled surfaces additionally introduce intensity variation into the diffuse component. So, although motion of the object does not disrupt the layout of the specular highlights, it does introduce variation in the peak intensities of the highlight regions. This means that, particularly at low specularities, intensity variation due to surface patterning can outweigh intensity variation due to specular reflection. The high levels of performance that we measure with marbled stimuli, even when they move, therefore argue against the suggestion that observers solve the operational colour constancy discrimination by tracking bright regions of the image. This is consistent with our findings in other experiments with static stimuli (see §4.C in [9], and [61]).

For marbled surfaces, the points that best distinguish illuminant from reflectance changes are those in which an intense region of the specular component of the image aligns with a dim region of the diffuse component, giving a chromaticity as close as possible to the illuminant chromaticity. The intensity variation in the diffuse component is higher for marbled stimuli than for bumpy stimuli, which may explain the overall performance advantage for marbled versus bumpy stimuli. For low specularities, the relative positioning of the diffuse and specular components can produce large variation in the availability of the illuminant chromaticity (see right panel of fig. 2 in [9]). So, motion of the marbled surfaces can indeed provide purer samples of the illuminant, because intense regions of the specular component might move over diffuse regions that have low intensity due to the surface pattern. However, at lower specularities, when there is only a small amount of chromatic variation between the diffuse and specular reflections, the temporal changes in lightness in the diffuse reflection on the regions on which the specular highlights lie might make distinguishing those chromatic differences more difficult. It may be the case, here and with respect to other cues, that any benefit to separating the diffuse reflection and specular highlights provided by the relative motion is counteracted by the local variability in lightness resulting from the same motion.

### 4.7. Motion and three-dimensional structure

We used separate conditions in which motion was introduced around the VA or around a PA specifically to test whether optic flow information about the three-dimensional test object improved operational colour constancy [44]. For matte stimuli, rotation around the VA would produce very similar proximal stimuli if the surface were a flat disc instead of a sphere (or bumpy sphere). However, rotation around any axis other than the VA provides kinetic-depth information and so a less-ambiguous sense of depth from the diffuse component should be evoked by the stimuli containing rotation around the PA. If constancy really is improved by such depth information we would expect better performance in the PA rotation conditions than the VA rotation conditions, but this is not what we find.

As motion and depth can sometimes afford similar information about three-dimensional shape, it is also useful to consider experiments that have manipulated binocular depth cues. Viewing three-dimensional objects with binocular depth information has been shown to give no advantage in operational colour constancy tasks [62,63]. However, binocular depth has provided an advantage in other colour constancy experiments where it may be used to segment the scene into different depth planes [64] or allow the interpretation of inter-reflections between surfaces [38]. In our previous study, also on operational colour constancy, we found that severely disrupting spatial cues to surface shape, by phase scrambling the image, did affect performance [9], and in other studies real stimuli do appear to enable better constancy assessed by a match-to-sample task [35]. For lightness constancy, motion that improved glossiness constancy (presumably by promoting perceptual separation of reflectance and shape properties) impaired performance on the lightness match [26]. So, although some cues to three-dimensional surface shape are important in judgements of surface colour and lightness, they can also introduce changes in the proximal stimulus that disrupt fine chromatic judgements.

In addition to providing information about the three-dimensional shape and material properties of the test object, motion may also provide additional information about the lighting environment. The smooth spherical surface of the marbled stimuli means that the light it reflects provides minimally distorted information about the light field in which the sphere sits. Indeed, the conditions in which the lights are stationary produce unchanging specular reflections, and motion of the object in these cases may provide particularly robust sampling of the light field, because variation in the diffuse component can be averaged out. Observers’ mean normalized performance showed no differences between motion conditions with marbled stimuli, but marbled stimuli produced better overall performance than bumpy stimuli.

### 4.8. Competing factors

Even for the simplified stimuli used in this study, and the performance-based task of operational constancy (that substantially removes the subjective bias that may be present in judgements of colour appearance), the sources of information that may influence the task are numerous, and their interactions complex. Competing factors may simultaneously enhance and impair performance in different conditions. However, we observe no effect of relative motion of surfaces and the lights that illuminate them, for all observers irrespective of their level of performance with static stimuli, and for all conditions. This implies that there are not consistent and separable effects of motion on perceptual scission and on disruption of local chromatic discriminations. If both factors are operating, their effects on performance must be closely coupled and opposite.

## 5. Conclusion

We systematically tested whether relative motion between light sources and a specular reflecting surface, and therefore relative motion of the diffuse and specular components of the image, affects discrimination of illuminant and reflectance spectral changes, either by promoting perceptual scission [65,66] between diffuse and specular components, or by impairing the fine chromatic discriminations that underlie operational colour constancy. We find no evidence that motion affects performance.

We compared two axes of rotation (to modify the available information about the three-dimensional nature of the test object that rotational motion provides), and compared bumpy and marbled objects (to introduce disruption or scrambling of informative image regions). We found no evidence of an effect of motion on either bumpy or marbled objects. Any benefit that the motion introduces may be counteracted by the increase in unpredictability of the locations of useful chromatic information. However, it is unlikely that these competing factors would perfectly cancel in all conditions, and for all observers. We cannot rule out the possibility of any benefit of motion in colour constancy for glossy objects, because we do not exhaustively test other variables associated with motion (speed, for example), or with object shape (curvature and complexity, for example), which may support better colour constancy. In addition, our stimuli were homogeneous in spectral reflectance and were rendered under a single spectral illuminant, rather than heterochromatic objects in complex lighting environments. A benefit of motion might be revealed by constructing stimuli that were purposefully ambiguous in the static case. However, we do not see an effect under the conditions of this experiment that have been carefully chosen to isolate the specularity cue to operational constancy and that require perceptual separation of the specular and diffuse components of the image.

Motion neither degrades nor improves performance irrespective of initial performance with static stimuli. We conclude, therefore, that motion is not instrumental in improving perceptual scission between diffuse and specular components of an image (because for the weakest observers, performance does not improve), and that motion does not lead to catastrophic failures of operational constancy, as one might have expected if performance were image based (because motion or disruption of informative image regions did not impair performance of the best observers, operating at the limits imposed by the chromatic statistics of the stimuli). As motion disrupts frame-by-frame low-level image statistics, the results imply that operational constancy with these stimuli depends on a high-level scene interpretation, which is maintained in all conditions.

## Supplementary Material

Raw results

## Supplementary Material

Spectra

## Data accessibility

The data generated in this study are available as the electronic supplementary material.
